# Preventive Applications of Polyphenols in Dentistry—A Review

**DOI:** 10.3390/ijms22094892

**Published:** 2021-05-05

**Authors:** Jasmin Flemming, Clara Theres Meyer-Probst, Karl Speer, Isabelle Kölling-Speer, Christian Hannig, Matthias Hannig

**Affiliations:** 1 Clinic of Operative Dentistry, Medical Faculty Carl Gustav Carus, Technische Universität Dresden, Fetscherstraße 74, D-01307 Dresden, Germany; Jasmin.Flemming@uniklinikum-dresden.de (J.F.); Christian.Hannig@uniklinikum-dresden.de (C.H.); 2 Special Food Chemistry and Food Production, TU Dresden, Bergstraße 66, D-01069 Dresden, Germany; karl.speer@chemie.tu-dresden.de (K.S.); Isabelle.Koelling-Speer@chemie.tu-dresden.de (I.K.-S.); 3 Clinic of Operative Dentistry, Periodontology and Preventive Dentistry, University Hospital, Saarland University, Building 73, D-66421 Homburg, Germany; matthias.hannig@uks.eu

**Keywords:** salivary pellicle, polyphenols, transmission electron microscopy (TEM), tea drugs, preventive dentistry, medicinal plants, molecular mechanisms

## Abstract

Polyphenols are natural substances that have been shown to provide various health benefits. Antioxidant, anti-inflammatory, and anti-carcinogenic effects have been described. At the same time, they inhibit the actions of bacteria, viruses, and fungi. Thus, studies have also examined their effects within the oral cavity. This review provides an overview on the different polyphenols, and their structure and interactions with the tooth surface and the pellicle. In particular, the effects of various tea polyphenols on bioadhesion and erosion have been reviewed. The current research confirms that polyphenols can reduce the growth of cariogenic bacteria. Furthermore, they can decrease the adherence of bacteria to the tooth surface and improve the erosion-protective properties of the acquired enamel pellicle. Tea polyphenols, especially, have the potential to contribute to an oral health-related diet. However, in vitro studies have mainly been conducted. In situ studies and clinical studies need to be extended and supplemented in order to significantly contribute to additive prevention measures in caries prophylaxis.

## 1. Introduction

Oral health is determined by a balance of commensal and pathogenic bacteria in the oral cavity. Here, commensal microbiota are dominant. Oral diseases are characterized by a breakdown of this homeostatic balance and result in a settlement of pathogenic bacteria [1]. From an economic point of view, oral diseases are a great burden for the health systems of many countries. They affect people over their complete live span. They are chronic and non-communicable diseases, and are often connected with factors such as smoking, bad oral hygiene, and alcohol abuse [2]. It is estimated that 3.5 billion people around the world suffer from oral diseases [3]. Caries is the number one reason for an oral disease in nearly 2.3 billion adults and more than 530 million children [3]. The expenses of caries treatment are not always covered by the governmental health services. Public health expenditure implies costs of around 5% and private health expenditures around 20% of the total costs. Although an early intervention and preventive measures are possible, it must be emphasized that the priority of prevention in general must increase. Through increasing urbanization, the lifestyle of people in low-income and medium-income countries changes. The advertising industry promotes the consumption of food and beverages with a high amount of sugar. In contrast, the awareness for oral health does not equally increase. Clearly, further development of preventive measures and health education must be emphasized.

Modern postindustrial nutrition contains high amounts of fermentable carbohydrates. In addition, a high proportion of acidic and sugar-containing foods and beverages is part of this type of nutrition. The teeth are protected by a physiological barrier provided by saliva under physiological conditions. This barrier is represented by a protective proteinaceous layer on the tooth surface (termed the acquired enamel pellicle). However, protection against demineralization via acids is not completely provided [4]. Preventive measures such as nutrition education, instructions of oral hygiene, and preventive and early therapeutic advances are necessary to put oral health and nutrition much more into the consumer’s focus [5]. The effect of these approaches is limited. In particular, the demographic change with an increase in life expectancy leads to topics such as age-adequate nutrition. Therefore, nutrition promoting oral health becomes central to dental research [6]. One of the main challenges in caries research is the increase in early childhood caries. Another important patient group is the group of multimorbid patients. The objective of food chemical analysis and nutritional science should focus on these specific groups. Main attention should be paid to the optimization of oral health and a prevention of age-dependent functional and physical degeneration. In this context, functional foods are increasingly becoming the focus of consumer attention. Functional foods fulfill basic functions such as providing a healthy diet and good nutrition. In addition, they contain health-related active components. These components are represented by bioactive ingredients such as polyphenols and promote oral and general health. The main goal is maintaining lifelong oral health.

Polyphenols belong to secondary phytochemicals, which are synthesized as protective and defense substances by plants. They contain a broad spectrum of molecules with at least one aromatic ring structure, different substitutes, and one or more hydroxyl group. Biological properties of polyphenols are antioxidative [7], anti-carcinogenic [8] and anti-inflammatory activities [9]. They are detected mainly in higher plants. Consciously or unconsciously, secondary phytochemicals are obtained via diet. The amount of ingested polyphenols is dependent on cultural and individual dietary habits. Plants used medicinally often contain high concentrations of polyphenolic compounds [10]. Based on their positive properties, polyphenols are of high interest for preventive dental science and novel approaches. These natural agents will be discussed in detail in the following sections. Previous reviews focused on the anticaries effects of polyphenolic compounds and their interactions with bacterial metabolic pathways, mainly reviewing in vitro studies [10,11]. This review represents a narrative overview of the current studies, focusing on the effects of polyphenols on the tooth surface, particularly the pellicle layer. Thereby, polyphenolic agents have been included that had been tested under in situ or in vivo conditions with potential clinical relevance for preventive dental medicine. Only a few selected in vitro studies were included to explain the specific mechanisms of action.

## 2. Polyphenols and Their Dietary Sources

Polyphenols are secondary plant compounds that occur almost ubiquitously in nature, with approximately 100,000 to 200,000 different metabolites known [12,13]. Depending on eating habits, polyphenols are taken in with daily food consumption to varying extents. They are found in vegetables, fruits, nuts, and legumes, and they are ingredients of polyphenolic beverages such as tea, coffee, and red wine [14]. Many reviews provide a good overview of the polyphenol content in the mentioned aliments (Table 1). Foods differ not only in their total polyphenol content, but also in their composition. Citrus fruits, for example, are very rich in flavones [15,16,17], and legumes such as soybeans contain almost exclusively isoflavones [18,19,20]. However, differences can also be found within one type of food. During cultivation, harvesting, and processing, the polyphenol content may vary. Depending on storage, the concentration and composition of the polyphenols can change [21]. During the maturation process, the concentration of anthocyanin increases while the concentration of phenolic acid decreases [21,22]. Changes in polyphenol content can also occur during cooking processes. Onions and tomatoes, for example, lose 75% of their quercetin content during cooking [23].

With a view to the low-sugar, mouth-healthy diet, it is particularly worth considering the various types of vegetables or tea.

## 3. Polyphenolic Tea Drugs in Dentistry

### 3.1. Classic Tea Drugs

The botanical name of the tea plant is camellia sinensis, an evergreen shrub belonging to the genus of the camellias, growing in tropical climates. Classic tea drugs are black, white, green, and oolong tea. They all originate from the leaves of camellia sinensis and they differ in their manufacturing process.

#### 3.1.1. Green Tea

Green tea is not fermented at all. Tea leaves are treated with steam or heated after harvesting. No enzymatic reaction can be observed compared to oolong and black tea. The flavor is determined by the selection of specific stems and leaves. The age of the leaves and the harvesting methods are thereby essentially significant.

Catechins are quantitatively dominant in green tea. They represent 15% of the dry mass. These catechins are epigallocatechin, epigallocatechingallate, and epicatechin [47]. Schlesier (2002) presumed that a major proportion of the antioxidative activity could be attributed to epigallocatechin, and the rest of the antioxidative activity come from epicatechin and from epicatechingallate. The amount of catechin decreases in the fermentation process. Consequently, green tea contains more catechins than fully fermented black tea.

#### 3.1.2. Oolong Tea

Oolong tea is a semi-fermented tea. During the manufacturing process, the tea leaves are only broken down at their edges. Consequently, the fermentation process is only partially undergone. Therefore, oolong tea contains less catechin than green tea. The catechins contained in oolong tea are epigallocatechine, epicatechine, epigallocatechin gallate, epicatechin gallate, gallocatachine, catechine, gallocatechin gallate, catechin gallate and epigallocatechin 3-*O*-(3-*O*-methyl) gallate [48,49,50,51,52,53,54].

#### 3.1.3. Black Tea

Black tea is completely fermented. After harvesting, the fresh tea leaves wither and become softer. Afterwards, they are rolled, and the cell walls of the leaves are broken down. Conclusively, a leakage of fluid can be observed. The fluids react with oxygen, and the polyphenol oxidase becomes activated. Through this process, a complete fermentation is achieved.

Through the fermentation process, 60% of the green tea catechins remain in black tea [55]. They react with the polyphenoloxidase to produce higher molecular theaflavines and theaflavingallate. Thereby, epigallocatechingallate is the most affected polyphenol during the oxidation process. Therefore, only 70% of the green tea epigallocatechin remains in black tea [47]. Moreover, black tea contains high concentrations of gallic acid. Gallic acid cannot be oxidized by polyphenoloxidase. Nevertheless, gallic acid can be oxidized to epitheaflavin acid by catechin–chinones. Due to the oxidation, the amount of theaflavines and theaflavin acid decreases and higher molecular thearubiginines are formed [56,57,58,59].

Overall, the concentration of catechins such as epigallocatechingallate, epicatechingallate and epicatechin decreases during the aging process of the tea leaves; thus, the concentration of theaflavins, thearubigin, and theabrownin increases [60]. Repeated tea infusion leads to a reduced amount of polyphenols overall; catechins are especially affected. In conclusion, multiple tea infusions reduce the antioxidative capacity of the tea.

### 3.2. Other Tea Drugs

Besides the classical tea drugs, black, green and oolong tea, the aim of the present study was to review the effect of different tea drugs containing high contents of polyphenols on the acquired enamel pellicle. Therefore, all available in situ and in vivo studies with effects on the pellicle layer were screened. Tea drugs with advantages for oral health were preselected, and single in vitro studies were chosen to explain distinct mechanisms involved in polyphenol–pellicle interactions.

#### 3.2.1. Cistus Incanus Tea

Medical properties of the evergreen shrubs of the genus cistus have been known for hundreds of years in the Mediterranean regions. Plant extracts from cistus incanus have been used to heal skin lesions. The reason is the antibacterial effect of cistus incanus.

*Cistus incanus* tea contains triple the amount of red wine polyphenols [61,62]. *Cistus incanus* polyphenols can be divided into three groups: ellagitannins (including gallic acid), flavanols, and flavonoles. Wittpahl et al. (2015) [63] determined the main elagitannins in *cistus incanus*. Punicalagin, punicalin, punicalagin-gallate and cornusiin B were thereby identified. Catechins and gallocatechines were determined in the flavanol group [64,65,66,67]. The six main flavanoles in *cistus incanus* tea are myricetin-galactoside, myricetin-pentoside, quercetin-galactoside, quercetin-xyloside, quercetin-rhamnoside, and tiliroside [68].

#### 3.2.2. Inula Viscosa Tea

The genus *Inula viscosa* (synonyme: dittrichia viscosa) originates from the family of composite plants (asteraceae) and contains more than 100 species [69]. This plant can be found in Africa, Asia and Europe, especially in the Mediterranean region. For hundreds of years, *Inula viscosa* has been used as a traditional folk remedy [69]. Positive properties are: antioxidative, anti-inflammatory, antiviral, antibacterial, antifungal, anti-carcinogenic, and antiallergic effects [69,70,71,72].

Brahmi-Chendouh et al. (2019) [73] examined the polyphenol profile of *Inula viscosa* with UHPLC-HR-MS/MS methods. They identified 43 polyphenolic compounds such as caffeic acid, caffeic acid derivates, 3,7-dihydroxycumarin, flavan-3-oles, quercetin glycoside, naringin and kaempferolglycoside [73]. In addition, the aglycones apigenin, naringenin, and luteolin were detected by other study groups [74,75,76,77].

#### 3.2.3. Fragaria Vesca Tea

*Fragaria vesca*, also known as wild strawberry, belongs to the genus *fragaria*, family rosaceae [78,79]. This plant can be found in moderate temperate zones in Europe and Asia [79]. *Fragaria vesca* is famous for its fruits, although the leaves and roots also contain high amounts of polyphenolic compounds [80,81,82].

A leaf extract from *fragaria vesca* contains 5–10% ellagitannines such as agrimoniin, pedunculagin, oligomere proanthocyanidines, ellagic acid, and flavonoles such as quercetin and kaempferol [83], as well as the anthocyanes anthocyanine and phenolic acids [78].

#### 3.2.4. Hamamelis Virginiana Tea

*Hamamelis virginiana* (witch-hazel) belongs to the family of hamamelidaceae [79]. These shrubs originate from the eastern part of North America. In addition, it is harvested in Europe. *Hamamelis virginiana* shrubs can reach a height of 7 m and bloom in autumn.

Bark extracts and leaf extracts of *hamamelis virginiana* are rich in hydrolysable and condensed tannins [79] and proanthocyanidines [84]. In the hydrolysable tannin fraction, hamamelitannin is dominant [85]. The proanthocyanidine fraction mainly contains catechin monomers and gallocatechin monomers, as well as procyanidin dimers. Hamamelitannin is a main component of the bark.

#### 3.2.5. Tormentil Tea

*Tormentil* originates from the genus potentilla and belongs to the rosaceae family. This herbaceous plant can be found in Central and North America and Europe [79]. Some smaller plant species were even discovered in tropical mountainous regions of South America [86]. The rhizomes have a bitter astringent taste and contain 20% tannins [86], ellagitannins, procyanidine, agrimoniin, a dimeric ellagitannin [87], 3-O-galloylquinic acid and catechins [88]. Additionally, *tormentil* has high concentrations of proanthocyanidines [89].

## 4. Metabolic Pathways in Higher Plants

**Figure 1:**
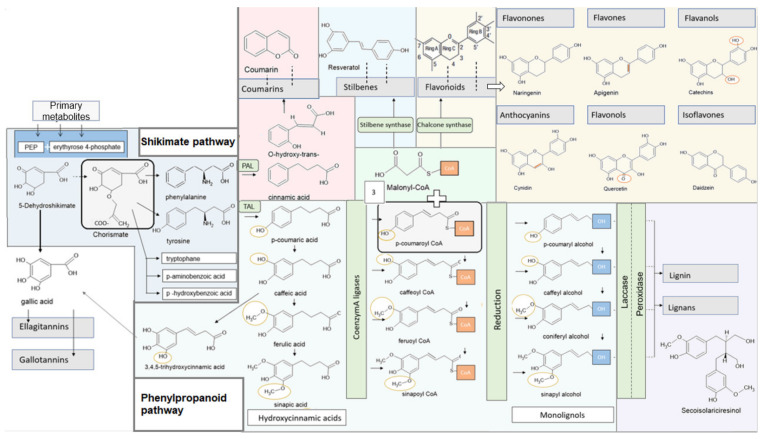
Illustration of phenylpropanoids synthesis and the synthesis of various polyphenols.

Phenols consist of an aromatic benzene ring with a hydroxyl group [90]. As soon as aromatic carbon compounds with two or more linked aromatic rings are generated, the substances are called polyphenols [91]. Plants have different metabolic pathways to synthesize different polyphenols. These pathways can be divided into a primary and a secondary metabolism. The primary pathways such as glycolysis, pentose–phosphate pathway, lipid–protein, and nucleic acid biosynthesis generate metabolites that are essential for plant survival [92]. However, plants also have the ability to secure biological benefits through the synthesis of various secondary plant metabolites. The plant synthesizes these components as natural deterrents against pests. The large variety of different secondary metabolites is caused by the phenylpropanoid metabolism [93] (Figure 1). It is named after the amino acid phenylalanine, which is usually the compound with which the biosynthesis begins.

In contrast to animals, plants have mechanisms to synthesize the amino acids phenylalanine and tryptophane themselves. Thus far, two pathways are known: the mevalonic acid pathway, and the shikimate pathway. In higher plants the shikimate pathway plays the major role [94,95].

### 4.1. Shikimate Pathway

The shikimate pathway is found in bacteria, fungi, and higher plants. It converts the primary metabolites phosphoenolpyruvate and erythyrose 4-phosphate via seven different enzymes to chorismic acid. The condensation reaction between erythrose 4-phosphate and phosphoenol pyruvate produces 3-dexoxy-d-arabino-heptulosonic acid-7-phosphate. It is then oxidized, dephosphorylated, and cyclized by dehydroquinate synthase. The resulting 3-dehydrochinate is dehydrated and then reduced to shikimate. Condensation with another phosphoenol, pyruvate, results in chorismate via the intermediate stage 5-O-enolpyruvyl shikimate-3-phosphate. This provides the precursor for the formation of the three amino acids tyrosine, phenylalanine and tryptophane, as well as p-amino and p-hydroxybenzoic acid (Figure 1) [94,95].

### 4.2. Phenylpropanoid Synthesis and Structure of the Individual Phenylpropanoids

The synthesis of polyphenols in plants is usually based on the amino acids phenylalanine and tyrosine. An exception is the formation of gallic acid, and therefore also the production of ellagitannins and gallotannins. They originate primarily from the shikimic acid pathway (Figure 1) [96]. Moreover, there are also a few plant phenols that are synthesized via a metabolic pathway called the polyketide pathway [97]

Phenylalanine ammonia lyase (PAL) is an enzyme responsible for the amination of phenylalanine to trans-cinnamic acid. Much less frequently, tyrosine is metabolized for the formation of phenylpropanoids. Tyrosine ammonia lyase (TAL) is responsible for the formation of tyrosine to p-coumaric acid [93]. Cinnamic acid and its derivatives are the basis for the synthesis of all polyphenols.

### 4.3. Specific Synthesis of Different Polyphenols

#### 4.3.1. Lignans, Lignin

Starting from the trans-cinnamic acid, the formation of different cinnamic acid derivatives can be distinguished. The formation of p-coumaric acid occurs through hydroxylation. By the methylation of p-coumaric, coffee acid can be formed and further methylation results in sinapin acid. These hydroxycinnamic acids can be activated by Coenzym A ligases (CoA ligases). Through reduction, it produces the corresponding alcohols, which are summarized under the term monolignols. This results in p-coumaryl alcohol, caffeyl alcohol, coniferyl alcohol, and sinapyl alcohol (Figure 1).

Monolignols themselves are toxic and unstable; thus, they are hardly detectable in living organisms. Glycosylation of the phenolic hydroxyl groups produces monolignol glycosides. In this state, the monolignols can be stored and transported within the plant. Two different enzymes, laccase and peroxidase, are responsible for the polymerization of the lignin alcohols to lignin and the lignans [98] (Figure 1).

#### 4.3.2. Gallic Acid, Hydrolysable Tannins: Ellagitannins, Gallotannins

For the formation of gallic acid two ways are are of particular importance [99]: Firstly via the phenylpropanoid pathway, where it is formed by the beta-oxidation of the 3,4,5-trihydroxycinnamic acid, which represents a further hydroxylation of the caffeic acid [100]; the second possibility results from the dehydrogenation of shikimic acid, which can proceed more immediately and is therefore considered the main synthesis pathway for gallic acid (Figure 1) [101].

It forms the basis for the synthesis of hydrolysable tannins, which include gallotannins and ellagitannins. They are esters of gallic acid with a polyol, such as 1-*O*-galloyl-β-d-glucopyranose. An increased substitution of the monoesters leads to 2-, 3-, 4-, 6-Penta-*O*-Galloyl-β-d-Glucopyranose. From this intermediate, ellagitannins can be formed by oxidative linkage with gallic acid; in gallotannins the galloyl groups are depsidically linked [102,103].

#### 4.3.3. Flavonoids, Stilbenes

The starting point for the formation of flavonoids and stilbenes is p-coumaroyl-CoA. Both pathways are linked with three molecules of malonyl-CoA from fatty acid metabolism [96].

The chalcone synthase forms tetrahydroxychalcone. This is normally converted by the chalcone isomerase directly to naringenin, a flavonoid [96]. Flavonoids consist of two benzene rings connected by a three-carbon linking chain. In addition, a pyran ring is formed centrally. Depending on the degree of oxidation of this ring, six different subclasses of flavonoids can be distinguished: flavanols, flavones, flavanones, flavanonols, anthocyanins, and isoflavones (Figure 1) [104].

The stilbenes are characterized by a skeleton with 1,2-diphenylethylene. They derive from the phenylpropanoid pathway and are formed by the reaction of the CoA esters of the cinnamic acid derivatives and three malonyl-CoA molecules with the stilbene synthase. Glycosylation, methylation, and prenylation lead to the formation of various complex stilbenes [105].

#### 4.3.4. Catechins and Theaflavins

Catechins and theaflavins are essential polyphenols of classical tea drugs. They belong to the flavonoid group. Their structure is characterized by two benzene rings that are linked by a heterocyclic pyran or pyron. At the position of the C3 carbon atom, the binding of gallate groups can occur through esterification [106].

This can result in various subgroups, such as catechin gallate (CG), gallocatechin (GC), epicatechin (EC), epicatechin gallate (ECG), gallocatechin gallate (GCG), epigallocatechin (EGC), and epigallcatechin gallate (EGCG) [107].

Fermentation can lead to the linking of catechins, resulting in the formation of theaflavins [108]. These are dimers of the catechins [109].

#### 4.3.5. Coumarins

The simple coumarins consist of a 1,2-benzopyrene skeleton. The synthesis starts with an ortho-hydroxylation of the cinnamic acid to o-coumaric acid. The product undergoes an isomerization from *cis*- to *trans*-formation. With the loss of a water molecule after lactonization, coumarin is formed (Figure 1) [110]. Various metabolites of simple coumarins are formed by hydroxyl, methoxyl, methylenedioxy, or isopentenyl groups. As soon as a furan or pyran ring is generated, the resulting coumarins are called furano- or pyranocoumarins. Dimers or trimers of the coumarins are called dicumarins; isomeric forms create isocoumarins [111].

## 5. Chemical, Physical and Biological Properties of Polyphenols

### 5.1. Chemical Properties

**Figure 2:**
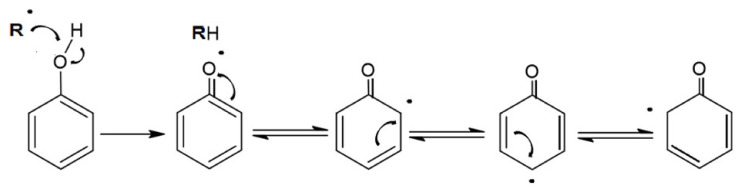
Illustration of the delocalization of electrons.

The characteristic structure of polyphenols determines their antioxidant properties [7]. Within the benzene ring, the carbon atoms are single-bonded. The remaining six electrons are free in movement. The hydroxyl group can also cause interactions with the electrons of the benzene ring. Within the conjugated aromatic ring, the unpaired electrons can be delocalized (Figure 2) and thus serve as a radical scavenger [96]. In addition, some phenolic compounds possess dihydroxy groups that can serve as metal chelators [96,97,98,99,100,101,102,103,104,105,106,107,108,109,110,111,112].

### 5.2. Physical Properties

Purified phenolic compounds are often white solids that have characteristic aromatic odors, such as vanillin, methyl salicylate, and eugenol [96]. There are some phenols that are more hydrophilic, such as the hydroxycinnamic acid glucosides, whereas others are more hydrophobic, such as polymethoxy flavonoids [96,113]. Hydrophobic compounds can be easily absorbed into the cell via diffusion. Various systemic pathways exist for the entry of hydrophilic compounds [96,114,115].

### 5.3. Biological Properties

These chemical and physical properties help to secure biological advantages for the plant. Lignin, as a complex polymer, is instrumental in three-dimensional structure formation. It helps to ensure mechanical transport and serves as a water barrier and water transport [116]. Particularly, flavones and flavonols play a role in protecting against UV light due to their absorption spectra. At the same time, the antioxidant properties of phenolic compounds help scavenge radicals that would cause damage from increased UV radiation [117,118,119]. Pigmented polyphenols serve as attractants for pollination of the plant [96,120]. The plant has different systems to defend itself from predators and pathogens [96]. Phenolic compounds often have antibacterial and antiviral activity themselves. Gallotannins and condensed tannins react strongly with various proteins and are thus also a decisive factor in the inhibition of enzymes [96]. Tannins, in particular, are also responsible for astringency and the bitter taste [121], which is intended as a protection against pests [96]. Some phenols even have a toxic effect to a certain extent [122].

## 6. Pellicle Formation and Initial Bacterial Colonization on Solid Surfaces

Soft and solid surfaces in the oral cavity are covered by a proteinaceous film called the pellicle [123,124]. Therefore, soft as well as hard tissues (enamel and dentin surfaces) are substrates for pellicle formation.

### 6.1. Mucosal Pellicle

Bioadhesion and -adsorption processes are significantly different on soft tissues compared to hard tissues. In contrast to oral hard tissues, oral epithelia represent shedding structures with high turnover rates. Thereby, outer layers of oral epithelia are covered with viable and dead cells. The formation of the mucosal pellicle is influenced by hydrophobic interactions and the cross-linking of proteins and salivary components [125,126,127,128,129]. Microorganisms can directly adhere to the epithelial cell membranes, whereas on the tooth surface, bacteria adhere indirectly through attachment to the acquired enamel pellicle layer [124,130,131,132,133]. The mucosal pellicle’s components are represented by soluble and epithelial mucins (mucin 5B, 7, 1, 4, 16) CA VI, sIgA, cystatin, amylase and PrPs [125,129,134,135,136]. Therefore, the mucosal pellicle represents an interface consisting of salivary and epithelial molecules [136]. As soon as polyphenols are consumed by food intake, they come into contact with the mucosal pellicle and modify the mucosal pellicle’s composition [137]. Nevertheless, a systematic examination of the influence of polyphenolic agents to the mucosal pellicle layer is difficult, due to the shedding character of this interface.

### 6.2. Acquired Enamel Pellicle

**Figure 3:**
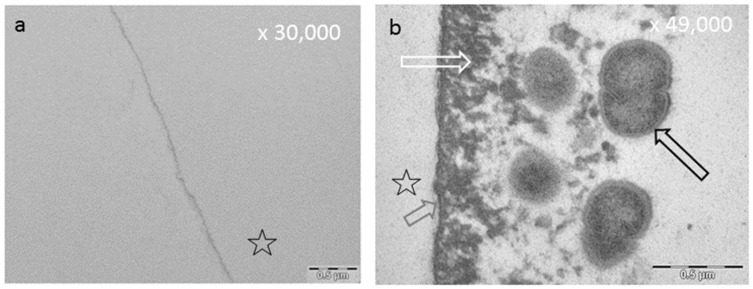
Representative TEM images of the 10 s (**a**) and the 24 h pellicle layer (**b**). The formation of a dense basal layer (**b**, grey arrow) can already be visualized after a few seconds. After 2 h, there are granular and globular structures (**b**, white arrow) which have absorbed onto this basal layer due to protein–protein interactions. As a result, an increase in pellicle thickness can be observed. (**b**) The result after 24 h biofilm formation. Please note the segmentation of the bacterial cell, indicating viability of the microorganisms (**b**, black arrow). Please note that all TEM images (Figure 3, Figure 4, Figure 5, Figure 7, Figure 8 and Figure 10) depict pellicles formed in situ. For this purpose, bovine enamel slabs were fixed to individual splints with silicone impression material for defined exposure to the oral fluids. The enamel was removed during the extensive sample preparation by acid etching; the former enamel site is marked with an asterisk. For details of the methods, please refer to Hertel et al. (2016) [77], Rehage et al. (2017) [68], and Kirsch et al. (2020) [149].

An acquired enamel pellicle is formed after a few seconds through the instantaneous adsorption of proteins, macromolecules (e.g., lipids), carbohydrates, and nucleic acids derived from saliva, gingival crevicular fluid, blood, bacteria, mucosa, and diet [138,139,140,141] (Figure 3). During pellicle formation in hard tissues and dental materials, two stages can be observed with transmission electron microscopy (TEM) [141,142]. The first stage shows a densely structured basal layer, reaching a thickness of about 10–20 nm [141,142,143,144]. The second phase of pellicle formation is characterized by protein–protein interactions leading to an outer pellicle layer with a globular appearance [144,145]. During the pellicle formation process, an increase in the pellicle thickness (100–1000 nm) can be observed after 60 min [141,143,146,147,148] (Figure 3).

There is high variability in pellicle protein profiles of different individuals [140,150,151]. The main functions are: lubrication of the enamel surface [133], mediation of de- and remineralization processes [123,152,153], and antibacterial functions [154,155,156]. The pellicle contains components that favor the adherence of oral bacteria. Bacteria have adapted to the physiological pellicle layer; thus, that the pellicle also promotes biofilm formation [157,158]. Specific receptors or ligands for the adhesion of bacterial species are glycolipids, fibrinogen, or collagen [158]. Pioneer bacteria such as different Streptococcus spp. or Actinomyces spp. are initially attached to the tooth surface via unspecific interactions [158,159,160]. Afterwards, they connect to the tooth surface irreversibly via specific receptors [161,162,163,164]. The number of adherent bacteria increases within the time of biofilm maturation. A cross-linking between the microorganisms of the biofilm can therefore be observed [165,166].

Within the consortium of caries-promoting bacteria, *Streptococcus mutans* is often utilized in in vitro studies to test specific anti-caries agents. For a long time, *S. mutans* was considered to be the most significant microorganism in the development of caries [167]. This bacterium is aciduric and has an acidogenic effect. *S. mutans* produces intracellular and extracellular polysaccharides [165,168]. Intracellular polysaccharides can act as the substrate supply for bacteria inside the three-dimensional biofilm structure. Extracellular polysaccharides are synthesized by bacterial glycosyltransferases (GTFs) [167]. The different isoenzymes of the GTF may differ in their properties. GTFs serve as specific receptors and are able to transform other bacteria into glucan producers [169]. Caries development has long been considered as a finely coordinated multifactorial process, in which the three-dimensional structure of the EPS matrix plays an important role [4].

The EPS matrix modulates the diffusion properties of the biofilm. Consequently, polymicrobial acid production is distributed spatially within the biofilm [170], and the hyperacidity within the biofilm structure is not buffered by saliva because the EPS matrix controls the transfer of salivary components [4]. Hence, dietary sugars can subsequently diffuse into the biofilm and salivary components are blocked [171,172,173]. The expressions of specific genes such as gtfB-genes, which are involved into the intake of carbohydrates, are upregulated if the host consumes a combination of sucrose and starch. This results in a complex conversion of the *S. mutans* transcriptome and regulates the dynamics of biofilm progression and pathogenicity [4,174,175]. This might be an explanation why combinations of sucrose and starch are more cariogenic than the individual substances [176]. This mechanism emphasizes the modulating effect of nutrition on the biofilm’s virulence. The pellicle is the starting point for biofilm formation on the tooth surface; therefore, anti-adherent and anticariogenic agents must attack this layer in order to reduce bacterial adherence.

### 6.3. Polyphenols as Anti-Adherent Agents

**Figure 4:**
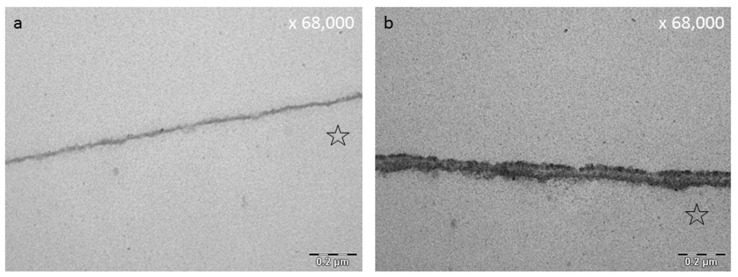
Effect of polyphenols on the pellicle’s ultrastructure, representative TEM images of the in situ pellicle after rinsing with tannic acid—oral exposure time 30 min: the control (**a**) shows a thin basal pellicle layer (Rehage et al. (2017) [68]; (**b**) a distinct increase in pellicle thickness and electron density after ex vivo rinsing with tannic acid (170 mg/mL, pH = 5.0). The former enamel site is marked with an asterisk.

**Figure 5:**
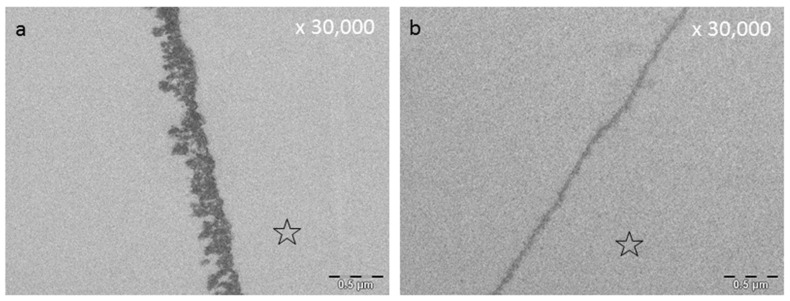
Both TEM images visualize the 2 h pellicle after a pellicle formation time of 1 min and rinsing with tannic acid for 10 min (**a**,**b**). (**b**) Sample generated after an additional treatment with HCl (pH 2.3) for 1 min in vitro (Hertel et al. (2017) [177]. In comparison, Figure 7a shows a 2 h control pellicle without rinsing. The former enamel site is marked with an asterisk.

External agents can modify the mucosa [137] and acquired enamel pellicle layer [68,77,149,177,178,179,180,181] (Figure 4), thereby bacterial adherence to the tooth surface is reduced [63,77,149,182] and the erosion-protective potential of the pellicle layer is enhanced [178,183] (Figure 5). Secondary phytochemicals such as polyphenols can act as such external agents.

Studies have shown that polyphenols already interact with salivary components in the oral cavity [184]. Typical salivary proteins for polyphenol-protein interactions are histatins and prolin rich proteins (PrPs). They also represent typical pellicle proteins [151,185,186,187]. It was shown in a recent proteome analysis that ECGC can increase the abundance of important pellicle proteins e.g., prolin rich proteins (PRPs), calcium-bind proteins and statherin [188]. One of the main mechanisms between PrPs and tannins derived from food intake is the so-called astringency. It describes a mouth puckering tactile feeling [121]. Astringency and bitter taste of food are strongly connected with flavor characteristics of a plant-based nutrition [121]. Often, consumers add milk to tea beverages to reduce astringency [121,189]. As a result, the bioavailability of secondary plant extracts is reduced, due to interactions between the polyphenols and the milk proteins.

Generally, astringency is attributed to the binding process between polyphenols and different classes of salivary components like PrPs, statherin and mucins [121]. PrPs are divided into basic, glycosylated and acidic PrPs. They provide the oral homoeostasis through the regulation of the calcium balance at the tooth surface [190,191]. Mucins are the main components of the mucosal pellicle and act as lubricants in the acquired enamel pellicle layer (e.g., mucin 7) [192]. Statherin represents an acidic salivary protein rich in tyrosin. Its main function is the regulation of the calcium and phosphate homoeostasis and the remineralisation of the enamel [121,193]. All these components are also integral parts of the acquired enamel pellicle. The interaction between these proteins and polyphenols are obtained by molecular forces, hydrophobic interactions and hydrogen bondings [194]. The connection of polyphenol-protein-complexes to the acquired enamel pellicle is probably based on crosslinking reactions leading to a resistant and hard to remove pellicle layer on the tooth surface [179].

**Figure 6:**
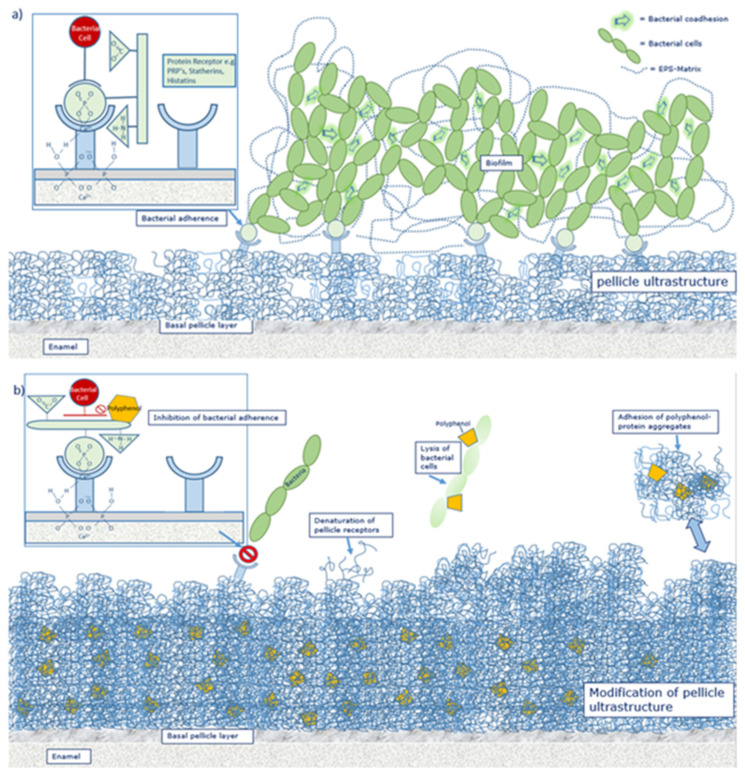
Denaturation of pellicle proteins through polyphenols. (**a**) Simplified representation of the pellicle ultrastructure on the tooth surface with an attached three-dimensional biofilm. Thereby, some proteins serve as receptors for bacterial cells to adhere to the tooth surface. (**b**) Hampered bacterial adhesion at the tooth surface. Polyphenols cause denaturation and changes in secondary and tertiary structure of proteins. In the following binding of bacteria to the polyphenol-modified-receptors is inhibited. Polyphenols also lead to a lysis of bacterial cells. In addition, polyphenol-protein aggregates adhere at the tooth surface. Consequently, the pellicle is denser and thicker in its ultrastructure.

Previous studies have also shown that polyphenols can denature pellicle proteins leading to an impaired interaction between microorganisms and bacterial receptors [182] (Figure 6). Thereby, the original structure of the receptor protein is broken down changing the bonding of the secondary, tertiary and quaternary structure. Especially plant polyphenols seem to have a tanning effect on the pellicle layer [63,141,179]. As a result, the pellicle is more electron dense and thicker as visualized by TEM [63,68,77,177,178] (Figure 4). Through the increase of pellicle-bound proteins, the pellicle becomes more protective against erosive attacks [178,183]. Besides, polyphenols have the ability to inhibit proteolytic salivary enzymes [62], matrix metalloproteinase, α-amylase and GTF [195,196]. Conclusively, glucan formation is reduced and less open binding sites for specific bacterial adhesion are available for cariogenic microorganisms [63,182,196,197]. Intentional interaction of plant substances with the acquired enamel pellicle are therefore a promising approach for caries prophylaxis and prevention of erosive loss of tooth structures [198].

## 7. Influence of Tea Polyphenols onto the Acquired Enamel Pellicle

### 7.1. Green Tea

**Figure 7:**
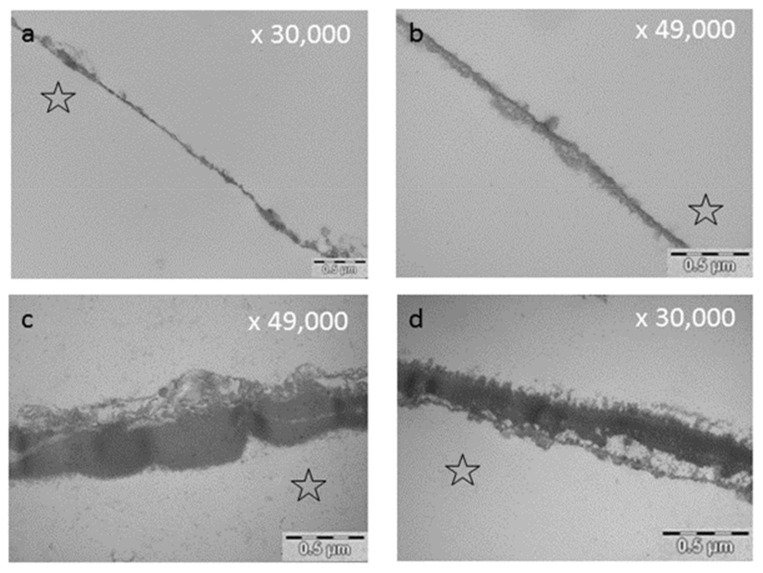
Representative TEM images of the pellicle ultrastructure after initial rinsing with different polyphenolic agents followed by 2 h of intraoral exposure. Rinsing with polyphenolic compounds leads to an increased pellicle thickness and density. The former enamel site was marked with an asterisk. (**a**) Native pellicle, control, no rinsing, intraoral exposure: 2 h, (**b**) Control, effect of CHX, intraoral exposure: 2 h, (**c**) Leaves of R. nigrum, watery plant extract 8 mL, 10 min rinsing, intraoral exposure: 2 h. For details consider Weber et al. (2015) [178], (**d**) Hamamelis (bark), 0.2 mg/8 mL, 10 min rinsing, intraoral exposure: 2 h. For details consider Kirsch et al. (2020) [149].

The influence of green tea onto the acquired pellicle was examined by Hannig et al. (2009) [182]. They observed a significant reduction of initial bacterial adhesion after 30 and 120 min of intraoral pellicle formation [182] (Table 2). Epigallocatechingallate is one of the main anti-oxidative polyphenolic compounds in green tea. In many studies EGCG is tested representatively as the main polyphenolic substance of green tea. Rehage et al. (2017) [68] demonstrated that rinsing with EGCG leads to an immediate increase in pellicle thickness and density (Table 2). The ultrastructure of the pellicle was therefore distinctly modified as shown via TEM (Figure 4, Figure 7). Further, EGCG-protein-aggregates accumulated at the pellicle surface leading to a fissured sectional image in TEM [68]. They were also detectable via REM [68]. Zimmermann et al. (2019) [181] confirmed these observations and showed that there was no influence of EGCG-rinsings on the negative charge of the in situ pellicle [181] (Table 2). Soares et al. (2020) [121] observed that flavanoles like EGCG and ECG highly interact with oral cells. In contrast, anthocyanes do not show such affinities.

### 7.2. Black Tea

Joiner et al. (2003) [180] examined the influence of black tea onto the acquired enamel pellicle. They showed that the pellicle structure was modified by adsorption of black tea components (Table 2). The modified pellicle could not be eluted by different buffer solutions. As a consequence, the removal, dissolution and replacement of these adsorbed components was strongly hampered under in vitro conditions [180]. It is assumed that the interactions of the black tea polyphenols lead to a cross-linking between the polyphenolic compounds and the pellicle as well as salivary proteins. In the following, pellicle formation is progressing. Whereby the polyphenol-modified pellicle layers are more resistant against removal than unmodified layers [179] (Table 2). This modification results in a doubling of the total protein amount binding to the hydroxy apatite surface in vitro [180] (Table 2). Further, the polyphenolic fractions of black tea contribute to this effect. Nevertheless, theaflavin, epigallocatechin and epigallocatechingallate showed less strong effects than the whole black tea extract. Pharmakokinetic investigations demonstrated that high concentrations of tea polyphenols, like theaflavin were still detectable in saliva after 1 h [199]. Theaflavin and thearubiginines are the main pigments of black tea. Ellipsometric studies showed that theaflavin is able to discolor the pellicle [179], which also might lead to stained teeth. Hannig et al. (2009) [182] observed the influence of black tea on the initial bacterial adhesion of the tooth surface after 30 and 120 min *in situ*. The combination of fluorescence methods (DAPI and FISH) clearly showed a significant reduction of initial bacterial adhesion after rinsing with black tea. Interestingly, black tea leads to a significant reduction of immobilized lysozyme activity after 3 and 30 min of oral exposition in situ [200] (Table 2).

### 7.3. Cistus Incanus Tea

The study of Wittpahl et al. (2015) [63] investigated different types of cistus incanus tea. They detected that especially the types rich in polyphenols had significant antibacterial effects in the *in vitro*-Live/Dead assay. After fractionation of the samples into seven fractions, only the fraction rich in ellagitannins and the flavonoid group had remaining weak antibacterial effects (Table 3). Simultaneously, the other fractions had no remaining effect at all [63]. Abdollahzadeh et al. (2011) [201] postulated that the in vitro antibacterial effects of cistus incanus against S. mutans are mainly attributable to hydrolyzable tannins and polyphenols with low molecular weight. On the other side, Naz et al. (2007) [202] report that polyphenols like quercetin, myrecetin and gallic acid are responsible for the antibacterial effect, whereas Hamilton-Miller et al. (2001) [203] and Otake et al. (1991) [204] assume that catechins inhibt the bacterial adherence to the tooth surface (Table 3). The study of Wittpahl et al. (2015) [63] shows in the in situ part of their examination that the antiadherent effect of cistus incanus cannot be attributed to single fractions of the polyphenolic plant compound but to the whole extract. Thus the ingredients of the whole extract display synergistic effects leading to a strong antiadherent and antibacterial effect. These findings have also been confirmed by the in situ study of Hannig et al. (2009) [182] (Table 3). Another in situ study showed in this context that cistus incanus tea had no impact on the immobilized pellicle enzymes [62] and detected no effect on the accumulation of lysozyme into the pellicle layer [198]. The underlying mechanisms are still not fully understood.

### 7.4. Inula Viscosa Tea

A recent in situ study demonstrated that 10 min-rinsing with *Inula viscosa* tea results in a significant reduction of the initial bacterial colonisation on the tooth surface after 8 h of oral exposition [77] (Table 3). Thereby, *Inula viscosa* tea exerts a significant antiadherent effect on oral streptococci [77]. Active polyphenolic substances in *Inula viscosa* tea are the flavonoid-aglycones apigenin, naringenin and luteolin. Their antibacterial effects on oral streptococci was observed in many in vitro studies [205,206,207,208] (Table 3). An animal study detected an inhibitory influence of apigenin on the glycosyltransferase isoenzymes [209]. Besides, different essential oil components of *Inula viscosa* tea seem to contribute to its antibacterial properties [210]. Though, the dominating antibacterial and antiadherent action is so far attributed to the polyphenolic compounds of *Inula viscosa* tea [77].

### 7.5. Fragaria Vesca, Hamamelis and Tormentil Tea

Kirsch et al. (2020) [149] examined the effect of the three polyphenolic tea drugs fragaria vesca, hamamelis and tormentil. They detected a prolonged antiadherent effect on bacterial colonization of the tooth surface as well as a distinct modification of the pellicle ultrastructure via TEM [149] (Figure 7). Thereby a thickening and an enhanced density of the ultrastructure was detected even after 8 h of oral exposition [149] (Table 3).

## 8. Mechanisms for Provided Effects by Polyphenols

### 8.1. Effect of Polyphenols on Oral Proteins

Besides their direct effect on bacterial cells, polyphenols exert an impact on the proteins of saliva and pellicle. Thereby, a so-called tanning effect can be observed [63,141,179,180]. The underlying principle is a denaturation of the receptor proteins functional groups resulting in a hampered bacterial adhesion (Figure 6) [182]. Polyphenols influence enzymes of the anabolism and catabolism of bacteria. It was shown that the human enzyme α-amylase and the bacterial isoenzymes glycosyltransferases are inhibited by polyphenols [196,197]. Both enzymes are part of the enamel pellicle and promote bacterial adhesion and biofilm formation.

**Figure 8:**
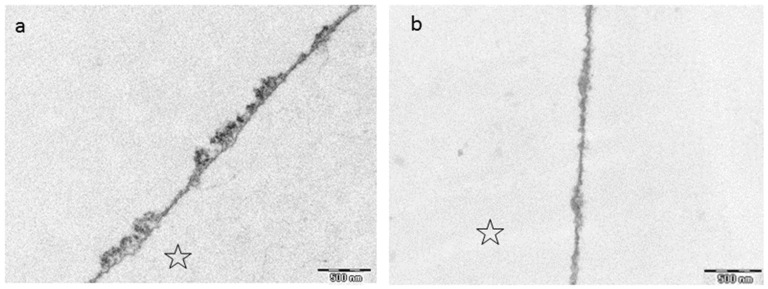
TEM images visualizing the effect of cistus incanus tea on the in situ pellicle layer after rinsing with 8 mL of cistus incanus tea for 10 min (Wittpahl et al. (2015) [63]). (**a**) shows the results after 30 min, (**b**) after 120 min. The former enamel site was marked with an asterisk.

It has been shown that bacterial glucan formation is reduced in situ after rinsing with hamamelis tea, fragaria vesca tea and tormentill tea [149]. Hence, it is assumed that rinsing with polyphenolic beverages leads to a reduction of binding sites for bacterial adherence on the tooth surface [203,204,212]. Interestingly, polyphenols from cistus incanus demonstrated no inhibition of the immobilized enzymes lysozyme, α-amylase and glycosyltransferase *in situ*, only peroxidase was affected [62]. The adsorption process of enzymes into the pellicle structure changes the secondary and tertiary structure of the specific enzymes [213]. As soon as the enzymes are integrated, the active site of the enzyme undergoes some chemical transformations and substantial conformational rearrangements of the proteins can be observed. Therefore, proteins that are originally soluble become insoluble through the adsorption to the pellicle layer. Nevertheless, the enzyme activity maintains [214,215,216] and can be enhanced [217] or reduced [218]. Due to these conformational changes and the reduction of free binding sites, polyphenols do not show the same impact on immobilized pellicle enzymes under in situ conditions compared to the in vitro situation. Through the immobilization process an inhibition of the enzymes (except peroxidase) is no longer possible. Consequently, the tanning effect of polyphenols does not affect all proteins of the acquired enamel pellicle [213], especially immobilized enzymes are excluded. Although, enzyme activities are not affected, polyphenols lead to a targeted protein enrichment of the pellicle layer. So far, all tested polyphenolic solutions showed an increase in pellicle thickness and denseness in in-situ-studies via TEM [68,77,149,177,178] or ellipsometric approaches [179,180] (Figure 4, Figure 7 and Figure 8). Consequently, the accumulation of specific proteins derived from saliva, gingival crevicular fluid, blood, epithelia and bacteria on the enamel surface is promoted and the proteins maintain their activity level [62]. This thickening effect has further tooth surface protecting effects (Figure 5). It was shown that tea drugs and plant extracts high in polyphenols such as black tea, green tea, Grape seed extract, Grapefruit seed extract, Origanum and leaves of the Ribes nigrum increase the erosion protective properties of the pellicle [178,183].

**Figure 9:**
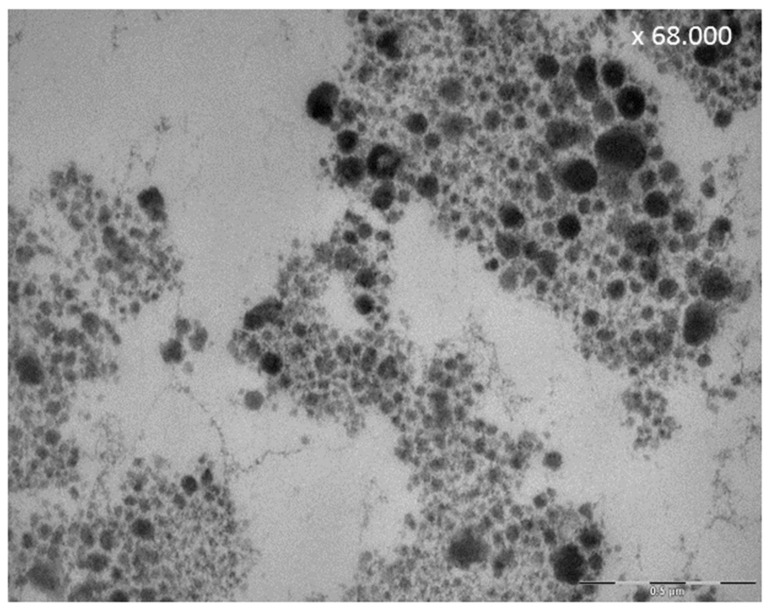
Effect of polyphenols on salivary proteins: Representative TEM-image of a saliva-sample after 30-s in vivo rinsing with 5% tannic acid, pH 2.0. The rinsing of tannic acid leads to aggregation of polyphenolic compounds with salivary proteins visible as electron dense globules.

Further, typical pellicle components like histatines and PrPs interact with polyphenols, building complexes in saliva and pellicle [185,186]. This is the reason for the astringent character of polyphenols [186]. Thereby the polyphenols can form insoluble aggregates with salivary proteins (Figure 9) [186], as shown by expectorate samples visualized by TEM [149].

At the pellicle layer these precipitates can also be found. The starting point are pellicle proteins with a high affinity to polyphenols, such as proline-rich proteins, amylases, cystatins and histatins. Afterwards interactions with the adsorbed glycoprotein layer can be observed [219]. On the chemical level different stages can be detected during the formation of polyphenol-protein-aggregates. First, soluble complexes are formed between the polyphenol’s aromatic rings and the pyrrolidine ring of the protein’s proline residues [220]. Second, insoluble complexes are built through cross-linking between peptides, and in the third stage, aggregates of complex precipitates separate spontaneously [221,222]. The cross-linking is described by two types of binding: hydrophobic interactions based on van der Waals-London interactions between the most apolar amino acids and the benzene rings of the polyphenols and on the other site hydrogen bonds [187,223,224]. As a consequence, more resistant pellicle layers are formed on the tooth surface, which are difficult to remove as shown for black tea [179]. These modified pellicle layers have higher acid-resistant properties than the physiological pellicle can provide [178,183] (Figure 5). Joiner et al. (2004) [179] assume that the prolin-rich proteins remain their polyphenol-binding site, so direct attachment of further polyphenols into the pellicle layers are possible. Covalent bonds play a subordinate role and are formed between the quinone forms of polyphenolic compounds (usually resulting from the oxidation of the latter) and the amino or thiol groups of proteins [225]. Hydrogen bonds, hydrophobic interactions and entropy are the driving factors of these processes between polyphenols and proteins [194]. External factors that influence the formation and the stability of the polyphenol-protein-aggregates are pH-value [226,227], ionic strength [227] and the presence of polysaccharides which can also disrupt the aggregation or rather modify the interaction processes [228]. Additional determinants for the stability of the aggregates are polyphenol ratio and concentration, temperature and the specific types of polyphenols and proteins [229]. Distinct differences can be observed between pellicle and salivary poteins since salivary proteins adsorb selectively to the pellicle layer [151]. The pellicle contains a wide range of protective proteins and enzymes from non-glandular sources enriched in the pellicle layer [150,155,181,230,231].

The interaction between polyphenols and proteins can lead to astringent sensations. This tactile feeling might be attributed to the degree of cross-linking between the adsorbed salivary proteins, polyphenols and epithelia of the oral cavity through transglutaminase enzyme reactions [129]. Nayak and Carpenter (2008) observed the polyphenol-protein-precipitates via TCA methods. Thereby, they detected mucin 1, 5a, 7, statherin and secretory components as the main protein components in these precipitates. In a proteome analysis statherin amounts were increased after the application of polyphenols (ECGC) [188]. These specific proteins are also components of the salivary and mucosal pellicle [184,232,233]. Consequently, these proteins are possible targets for intentional modulation of the pellicle. Further research, like complex proteome analysis are necessary to give further proof.

### 8.2. Effect of Polyphenolic Compounds on Bacteria

Most of the assumed reasons for antiadherent and antibacterial effects of polyphenols have been derived from in vitro studies [70,197,203,204,234,235,236,237,238,239,240]. Thereby, it was shown that polyphenols can break trough bacterial cell walls and interact with intracellular membranes [63]. Further, the chemical character of polyphenols results in complex formations with metal ions. Consequently, the essential metal ions are no longer available for microorganisms leading to a reduction of bacteria [11,63,202,241].

The effect of tea polyphenols to cariogenic bacteria was mainly examined under in vitro conditions. Karygianni et al. (2014) [242] detected the antibacterial properties of *Inula viscosa* tea against nine relevant oral microorganisms. Thereby, *Inula viscosa* tea showed strong antibacterial effects against *S. mutans, S. sobrinus, S. oralis, E. faecalis, Candida albicans, E. coli, Staph. aureus, Porphyromonas gingivalis, Prevotella intermedia, Fusobacterium nucleatum* and *Parvimonas micra* [242]. The study group detected that a methanolic solution of *Inula viscosa* tea was more effective than an ethyl acetate extract [242]. Besides, Xiao et al. (2000 a, b) [243,244] examined that tea polyphenols can inhibit the adherence of cariogenic bacteria like *S. mutans* and *A. viscosus* to hydroxyapatite covered with saliva or collagen *in vitro*. Other in vitro studies investigated the inhibition of GTF-dependent glucan synthesis by specific polyphenols [197,245,246,247,248,249]. It is assumed that only the polymeric fraction of tea polyphenols is responsible for this inhibiting activity [247,248]. Besides, three studies relate the inhibiting effect of acid production by mutans streptococci with the inhibition of the bacterial enzyme proton translocating F-ATPase [245,246,250]. Thereby, bacterial growth [245,250] and adhesion to hydroxyapatite is inhibited [243,244]. In addition, it is assumed that microorganisms that are exposed to polyphenols up-regulate proteins that modulate the defense mechanisms of the bacterial cell and down-regulate metabolic and biosynthetic proteins that are involved in amino acid synthesis, protein formation and energy metabolism [205,251]. Consequently, glucan formation is also down regulated as shown in the in situ study by Kirsch [149]. A distinct influence on the 3-dimensional structure of the biofilm is thereby possible, in addition available binding sites and the pathogenicity of the biofilm might be influenced.

### 8.3. Effect of Tea Polyphenols on the Ultrastructure of the Pellicle

The pellicle layer represents a 0.1–1 μm thick protein layer on the tooth surface [138]. The defined two-layer structure of the pellicle consists of an electron-dense basal layer and a less electron dense second layer with granular and globular structures as visualized by TEM [124,141] (Figure 3). It was shown that the pellicle ultrastructure can be enhanced by plant extracts high in polyphenols, such as Origanum and leaves of Ribes nigrum (Figure 7) [178].

Tea polyphenols can modify the pellicle layer as well. A pellicle thickening and enhancing effect can be observed [179] (Figure 3, Figure 4, Figure 7 and Figure 8). Rehage et al. (2017) [68] showed that rinsing with EGCG can enhance the pellicle thickness under in vitro conditions. This effect is caused by the integration of protein-polyphenol-aggregates with an average diameter of 70 nm. Consequently, the pellicle layer obtains a fissured appearance in TEM images [68]. Further, rinsing with EGCG under in situ conditions leads to a more homogenous appearance of the pellicle structure. Also under in situ conditions, electron dense, aggregated structures can be detected [68]. These structures consist of ECGC-protein-aggregates. In the in situ study, the pellicle was not as fissured as under in vitro conditions. Consequently, ECGC-molecules and salivary proteins already connect in the salivary film of the oral cavity, leading to the assumption that the effective concentration of the tea polyphenols that connects with the tooth surface is reduced significantly [68].

Similar effects were shown for *Inula viscosa* tea. After rinsing a thicker more electron dense pellicle layer was observed [77]. Round electron dense complexes mit embedded vacuoles above the pellicles basal layer were detected by TEM [77]. *Inula viscosa* contains hydrophobic components such as essential oils (nerolidol and linalool) [77,210]. These hydrophobic lipid components may cause the observed vacuoles inside the modified pellicle structure [77].

Kirsch et al. (2020) [149] examined the effect of fragaria vesca, hamamelis and tormentil tea on the ultrastructure of the pellicle. In their study, a modification of the ultrastructure of the pellicle was still detectable after 8 h of oral exposition. Similar effects of these specific polyphenols on the ultrastructure were visible. A significant enhanced and more electron dense pellicle ultrastructure with polyphenol-protein aggregates was visualized by TEM [149] (Figure 7). In their study, the pH of the phenolic tea drugs differed (fragaria vesca: 5.53, tormentil: 5.28, hamamelis: 4.3). Therefore, zones of demineralization were detected after rinsing with hamamelis. These areas were infiltrated and covered with pellicle compounds. The authors thereby assume a repair process of superficial erosive defects filled with proteinaceous structures. On top of these structures a granular surface pellicle has formed, leading to repair processes. These areas are known as subsurface pellicle and they can be identified in the enamel [149,252]. It would be very interesting to examine the antiadherent properties of the polyphenolic tea drugs at a higher pH-value to prevent initial demineralization of the tooth surface.

In summary, polyphenolic tea drugs lead to a distinct modification of the pellicle layer as shown by TEM in multiple studies. The extent to which a modification can be detected depends on the polyphenolic tea drug.

### 8.4. Influence of Tea Polyphenols on Erosive Attacks of the Enamel Surface

A recent proteomic analysis showed that the treatment with EGCG gel enriches proteins with potential functions to protect against caries and erosion [188]. Thereby the abundance of important structural and functional proteins was increased (e.g., PRPs, calcium-bind proteins and statherin). Additionally, the EGCG-modified pellicle proteins were examined after citric acid attacks [188]. The result was an increase in cystatins and profilin-1 [188]. Hertel et al. (2017) [177] simulated alimentary acid attacks by incubation in HCL and examined whether polyphenols have an erosion-protective effect on the pellicle layer and the enamel surface [77] (Figure 5). Erosive conditions were simulated with pH-values 2.0, 2.3 and 3.0. After rinsing with *Inula viscosa* tea a loss of the pellicles basal layer and pore volume and density of the enamel surface was detected after 30 min and 120 min oral exposition [77]. Thus, *Inula viscosa* tea provides no erosion protective potential for the pellicle covered tooth surface [77]. In contrast rinsing with a combination of watery plant extracts of Origanum and Ribes nigrum leaves lead to a more electron dense and thicker pellicle layer with an enhanced erosion protective potential of the pellicle layer and therefore a protection of the enamel surface against erosive attacks [178]. These results were confirmed by another in vitro study that examined the effect of different polyphenol-rich teas and natural extracts on the erosion –protective properties of the pellicle [183]. This study outlines that not all teas high in polyphenols have beneficial effects. Black tea and green tea, as well as grape seed extract and grapefruit seed extract seem to have pellicle-modifying abilities and improved its protecting effect against enamel erosion under in vitro conditions [183].

## 9. Discussion

For conventional prevention, the use of fluoride is recommended. Studies show that regular fluoride intake can limit but not completely prevent the development of caries [253]. Biofilm removal and oral hygiene measures, as well as the additional influence of fluoride, can only alleviate this problem [254]. The antiseptical solution chlorhexidine is used postoperatively for prophylaxis. It has a very good antibacterial efficacy, but is insufficiently effective against bacteria already bound to the tooth surface [255,256]. The cationic character of the chlorhexidine molecules is responsible for the effect on the negatively charged bacterial surface. At the same time, however, this structure also causes severe discolouration, dry mouth, irritations of flavour and a shift of the bacterial balance in the oral cavity. Therefore, CHX is not suitable for daily use [257].

The formation of antibiotic resistance is also a problem in the treatment of infectious diseases caused by bacteria. There are more than 700 microbial species in the oral flora [258]. If bacteria are already present in a biofilm structure with an extracellular matrix, resistance is 1000 times more likely than among planctonic bacteria [4].

**Figure 10:**
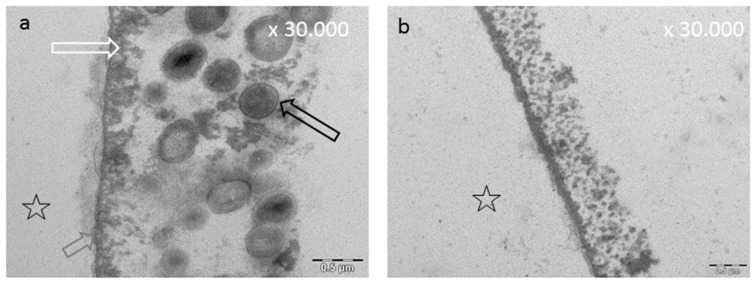
Sustainable long-term effects of tannic acid on bioadhesion and biofilm formation in situ, TEM. After 24 h of oral exposure, bacterial biofilm formation was observed (**a**,**b**) as also described in Xi et al. (2020) [259]. (**a**) shows an image of the control without rinsing. The basal pellicle layer is marked with a grey arrow, the globular pellicle layer is marked with a white arrow and the bacterial cell is marked with a black arrow. Tannic acid modified pellicle is covered by bacteria and extracellular biofilm matrix (**b**). The volunteers rinsed with a solution containing 1% tannic acid and 1% chinese gallnut extract, 2 times a day. The first time 3 min after the splint was inserted, the second time before night sleeping. As also seen in Figure 9, aggregates enriched from the saliva can be observed above the pellicle. The former enamel site was marked with an asterisk.

Polyphenols are secondary plant compounds associated with antibacterial, anti-inflammatory and anti-carcinogenic properties [7,8]. The current body of studies shows that polyphenols lead to thickening and strengthening of the pellicle layer [68,77,179], improving the pellicles erosion-protective potential [178,183]. In addition, polyphenols show efficacy in inhibiting bacterial growth [149,242,243,244], adhesion of bacteria to the tooth surface [149,182,259] and inhibition of enzyme activity [196,197]. Consequently, there is a further reduction of receptors that can serve as targets for the adherence of bacteria [203,204,212] and reduced glucan formation in situ [149]. So far, there are only two studies that examined the effect of polyphenolic tea fractions onto the acquired pellicle [63,141]. The comparison of the effect of the total extract versus fractions high in specific polyphenols highlight that complete extracts of specific teas have a stronger antibacterial effect than the specific fractions [63,141]. Besides investigating the total extract or fractions of polyphenol plant extracts, some studies analysed the effect of individual substances like epigallogcatechingallate or tannic acid [177,259,260]. Thereby, tannic acid is a good example to illustrate the mode of action and the long-term effect of polyphenols onto the pellicle layer [177,259,260]. So far, pure polyphenolic substances are not available for customers, due to the high costs for the production of pure substances like tannic acid. A closer look at the effect of tannic acid reveals a good efficacy on short-term pellicles (30 min, 120 min and 8 h). A study by Xi et al. (2020) [259] could additionally observe that tannic acid still exhibits anti-adherent properties after 24 h (Figure 10). Schestakov et al. (2020) [260] even detected anti-adherent efficacy after 48 h, which is similar to the effect of chlorhexidine and therefore of particular clinical relevance. The effects of tannic acid underline the importance to gain further knowledge about polyphenols since they provide long-lasting effects, which could be beneficial for preventive oral health care.

Overall, most of the currently available studies are mainly based on in vitro conditions. Further studies, especially in vivo and in situ studies, are necessary to evaluate possible interactions with long-term medication. Besides, it is necessary to understand the interactions of polyphenols with other surfaces within the mouth (mucosal pellicle, tongue) in order to reduce the total number of oral bacteria and minimise recolonization of the teeth. Bioadhesion is not only limited to the tooth surface (acquired enamel pellicle), these phenomena occur at all surfaces in the oral cavity. However, there are differences in adsorption since oral soft tissues constantly change through desquamation. Therefore the mucosal pellicle is an own entity. Interactions between polyphenols and components of the mucosal pellicle can be observed [137]. Polyphenols (tannins) react with mucins (mucin 5B) to mucin 5B-tannin-precipitates and their size increase with higher tannin concentrations and galloyalation [137]. In presence of basic PrPs, the size and distribution of these polyphenol-mucin-aggregates is altered [137]. In this context, promising results from a clinical trial suggests the use of cistus incanus tea in patients with mucositis or treatment-related radiation-induced oral mucositis [261]. Thereby, cistus incanus had an effect on the occurrence of mucositis regarding latency and incidence [261].

## 10. Conclusion and Perspectives

Although polyphenolic teas and beverages do not completely prevent bacterial adherence to the tooth surface but some of them reduce it significantly over a period of time [149]. Therefore the authors recommend the use of this tea, high in polyphenolic components, in addition to intensive oral care and hygiene along with the application of fluorides [261]. Especially patients with oral mucositis can benefit from the oral application of polyphenols [261].

Future studies should include further polyphenolic tea beverages to gain more basic knowledge about polyphenolic interactions within the oral cavity. Especially studies regarding the influence of specific polyphenols on the pellicle microbiome and proteom should be subject to further investigations.

Overall, polyphenolic teas and substances are relevant biological approaches for preventive strategies. Nevertheless, the most important part in preventive measures is still mechanical biofilm management where cleaning with a toothbrush and the use of fluorides remain essential parts of oral health. Polyphenols can be considered as important additives to a tooth-friendly and healthy diet.

## Figures and Tables

**Table 1:** Food and beverages with high polyphenolic contents.

Food or Beverage		References
**Vegetables**	Different Tomato Varieties	[24,25,26]
	Pepper	[25]
	Eggplant	[25]
	Olive Pomace	[27]
	Potato	[25,26]
	Lettuce, Onion	[26]
**Fruits**	Fruit and Fruit Juices	[28,29]
	Different Fruits	[30]
	Pomegranate	[31,32]
	Mango	[33]
	Strawberry	[34,35,36]
	Blueberry	[34,35,36]
	Raspberry	[35]
	Blackberry	[35]
	Cranberry	[35]
	Cherry	[26]
	Apple	[26]
**Nuts**	Raw Nuts	[37,38]
**Legumes**	Beans	[39]
	Chickpea, Green Gram, Pearl Millet, Finger Millet	[40]
**Tea**	Black Tea	[41,42,43,44]
	Green Tea	[41,42,43,44,45]
	White Tea	[45]
**Coffee**		[41,44]
**Wine**	Red and White Wine	[46]

**Table 2:** Comparison of different tea polyphenols and their effect on the tooth surface.

Tea Variety	Polyphenol	Effect	Reference	Study Design
Green Tea	EGCG	- Increase in pellicle thickness and density	[68]	In situ
		- Increase in pellicle thickness and density - No chance of charge of the tooth surface	[181]	In situ
	Whole Green Tea	- Significant reduction in initial bacterial adhesion after 30 and 120 min of oral exposure	[182]	In situ
Black Tea	Whole BlackTea, ECG, EGCG, Theaflavin	- Absorption of ECG, EGCG, theaflavin on the tooth surface, modification of the in-vitro pellicle	[179]	In vitro
		- Modification of pellicle structure due to cross-linking between polyphenolic compounds and the pellicle as well as salivary proteins	[180]	In vitro
	Catechins, Theaflavins	- Binding to salivary	[199]	In vitro, in vivo
	Whole Black Tea	- Significant reduction in the initial bacterial adhesion after 30 and 120 min	[182]	In situ
		- Significant reduction in immobilized lysozyme activity after 3 and 30 min	[200]	In situ

**Table 3:** Comparison of different tea polyphenols and their effect on the tooth surface.

Tea Variety	Polyphenol	Effect	Reference	Study Design
Cistus Incanus	Ellagitannins, Flavonoid Group	- Antibacterial effects In-vitro-Live/Dead assay - In situ: Reduced initial bacterial colonisation	[63]	In vitro, in situ
	Hydrolysable Tannins	- Antibacterial effect against S. mutans	[201]	In vitro
	Quercetin, Myrecetin, Gallic Acid		[202]	In vitro
	Catechins	- Prevention of bacterial adhesion - Inhibition of GTF - Inhibition of amylase	[203], [204]	In vitro
	Whole Extract	- Significant reduction in the initial bacterial adhesion after 30 and 120 min	[182]	In situ
		- No impact of lysozyme and the immobilized enzymes	[62], [198]	In situ
Inula Viscosa	Flavonoids	- Antibacterial activity against cariogenic bacteria	[211]	In vitro
	Luteolin	- Antimicrobial effect	[206]	In vitro
	Apigenin	- Antibacterial activity	[208]	In vitro
			[209]	In vivo
	Whole Extract	- Antiadherent effect - Significant reduction in adherent bacteria	[63], [77], [182]	In situ
Fragaria Vesca, Hamamelis, Tormentil		- Prolonged antiadherent effect on bacterial colonization, thickening and an enhanced density of ultrastructure	[149]	In situ

## Data Availability

Not applicable.
